# Monitoring, prophylaxis, and treatment of infections in patients with MM receiving bispecific antibody therapy: consensus recommendations from an expert panel

**DOI:** 10.1038/s41408-023-00879-7

**Published:** 2023-08-01

**Authors:** Noopur Raje, Kenneth Anderson, Hermann Einsele, Yvonne Efebera, Francesca Gay, Sarah P. Hammond, Alexander M. Lesokhin, Sagar Lonial, Heinz Ludwig, Philippe Moreau, Krina Patel, Karthik Ramasamy, Maria-Victoria Mateos

**Affiliations:** 1 Division of Hematology and Oncology, Massachusetts General Hospital, Boston, MA USA; 2 Department of Medical Oncology, Dana-Farber Cancer Institute, Boston, MA USA; 3 Department of Internal Medicine II, University Hospital Würzburg, Würzburg, Germany; 4 Division of Blood and Marrow Transplant, OhioHealth, Columbus, OH USA; 5 Division of Hematology 1, Clinical trial Unit, AOU Città della Salute e della Scienza di Torino, University of Torino, Turin, Italy; 6 Division of Infectious Diseases, Massachusetts General Hospital, Boston, MA USA; 7 Memorial Sloan Kettering Cancer Center, New York, NY USA; 8 Weill Cornell Medical College, New York, NY USA; 9 Winship Cancer Institute, Emory University School of Medicine, Atlanta, GA USA; 10 Wilhelminen Cancer Research Institute, Department of Medicine I, Center for Oncology, Hematology and Palliative Care, Klinik Ottakring, Vienna, Austria; 11 Department of Hematology, University Hospital of Nantes, Nantes, France; 12 Department of Lymphoma and Myeloma, Division of Cancer Medicine, The University of Texas MD Anderson Cancer Center, Houston, TX USA; 13 Oxford University Hospitals, Oxford University, NHS Foundation Trust, Oxford, UK; 14 Radcliffe Department of Medicine, Oxford University, NHS Foundation Trust, Oxford, UK; 15 Department of Hematology, University Hospital of Salamanca, IBSAL and Center for Cancer Research, Salamanca, Spain

**Keywords:** Disease prevention, Myeloma, Drug development

## Abstract

Bispecific antibodies (BsAbs) are emerging as an important novel class of immunotherapeutic agents for the treatment of multiple myeloma (MM), and are set to be more widely used in clinical practice. However, this new class of therapies is associated with a distinct adverse event (AE) profile that includes cytokine release syndrome and immune effector cell-associated neurotoxicity syndrome, as well as AEs leading to increased infection risk such as cytopenias and hypogammaglobulinemia, and infections themselves. As preliminary data with this class of agents shows an increased risk of infections as compared with conventional MM treatment regimens, such as immunomodulatory drugs, proteasome inhibitors, and anti-CD38 monoclonal antibodies (mAbs), guidance on infection monitoring, prophylaxis and treatment is required. This review provides consensus recommendations from a panel of 13 global experts, following a meeting in August 2022. The meeting objective was to review existing literature and identify relevant information on infections with all BsAbs in patients with MM, as well as to discuss clinical experience of experts in managing these infections. The recommendations outlined here can be used to guide management of infection risk factors, such as hypogammaglobulinemia and neutropenia. In addition, they can be used to guide the monitoring, prophylaxis, and treatment of bacterial, viral and fungal infections, including emerging infections of interest, such as coronavirus 2019 (COVID-19), and the use of vaccinations prior to and during BsAb treatment. The recommendations have been graded by the panel based on level of data available. Key recommendations include universal herpes simplex and varicella zoster virus prophylaxis, screening for hepatitis B virus reactivation risk in all patients, monthly intravenous immunoglobulin treatment for immunoparesis and in the absence of life-threatening infectious manifestations, use of colony-stimulating factors in patients with Grade 3 neutropenia, universal *pneumocystis jirovecii* pneumonia prophylaxis and no routine anti-fungal prophylaxis.

## Introduction

Bispecific antibodies (BsAbs) are an emerging novel class of immunotherapeutic agents for the treatment of multiple myeloma (MM) [1]. BsAbs act by binding to two targets, one on tumor cells and one on effector T cells, allowing the formation of an immunological synapse, resulting in T cell activation and thereby tumor cell lysis [2–4]. Current BsAbs under investigation target a variety of MM-specific antigens, including B cell maturation antigen (BCMA), a target for which agents with different mechanisms of action are available, such as antibody-drug conjugates (ADC) and chimeric antigen receptor (CAR) T-cell therapies. A number of BsAbs are currently being evaluated in various MM settings (Table 1). Teclistamab and elranatamab are the BsAbs targeting BCMA which are the furthest in development. Teclistamab has received accelerated approval from the European Medicines Agency (EMA) for the treatment of adult patients who have received ≥3 prior therapies including an immunomodulatory agent, a proteasome inhibitor (PI), and an anti-CD38 antibody [5], and has received accelerated approval by the U.S. Food and Drug Administration (FDA) for the treatment of adult patients with relapsed/refractory MM (RRMM), who have received ≥4 prior therapy lines [6]. Elranatamab has received priority review and breakthrough therapy designation by the FDA and EMA for RRMM [7, 8]. Talquetamab, a G protein-coupled receptor family C group 5 member D- (GPRC5D) directed BsAb, and cevostamab, a Fc receptor-like protein 5 (FcRH5) directed BsAb are also being developed for RRMM [9, 10].

**Table 1:** Summary of BsAbs in development for MM.

BsAb	Target	Development stage	Ongoing studies		
			Study	Monotherapy or combination	Patient population
Teclistamab	BCMA x CD3	EMA approved for adult RRMM who have received at least three prior lines of therapy (including an IMiD, PI, and anti-CD38 antibody) [5] FDA approved for adult RRMM patients who have received four prior lines of therapy (including a PI, IMiD, and anti-CD38 monoclonal antibody) [6]	MajesTEC-1 (NCT04557098) [24]	Monotherapy	RRMM
			MajesTEC-2 (NCT04722146) [77]	Combination with anti-cancer therapies	MM
			MajesTEC-3 (NCT05083169) [78]	Combination with daratumumab	RRMM
			MajestTEC-4 (NCT05243797) [79]	Combination with lenalidomide	NDMM (as post-ASCT maintenance)
			MajestTEC-7 (NCT05552222) [80]	Combination with daratumumab and lenalidomide	NDMM who are transplant ineligible or not intended for ASCT
			MajesTEC-9 (NCT05572515) [81]	Monotherapy	RRMM
			NCT04696809 [82]	Monotherapy	RRMM
			IFM 2021-01 (NCT5572229) [83]	Combination with daratumumab or lenalidomide	MM (elderly patients)
			RedirecTT-1 (NCT04586426) [84]	Combination with talquetamab	RRMM
			TRIMM-3 (NCT05338775) [85]	Teclistamab or talquetamab in combination with PD-1 inhibitor	RRMM
			ImmunoPRISM (NCT05469893) [86]	Monotherapy	Smoldering MM
			MASTER-2 (NCT05231629) [87]	Combination with daratumumab	NDMM
			NCT04108195 [88]	Teclistamab/talquetamab combination with daratumumab and/or pomalidomide	MM
Elranatamab	BCMA x CD3	Phase III	MagnetisMM-1 (NCT03269136) [89]	Monotherapy or in combination with lenalidomide or pomalidomide	RRMM
			MagnetisMM-2 (NCT04798586) [90]	Monotherapy	RRMM
			MagnetisMM-3 (NCT04649359) [91]	Monotherapy	RRMM
			MagnetisMM-4 (NCT05090566) [92]	Combination with either nirogacestat or lenalidomide + dexamethasone	RRMM
			MagnetisMM-5 (NCT05020236) [93]	Monotherapy and in combination with anti-cancer therapies	RRMM
			MagnetisMM-6 (NCT05623020) [94]	Combination with daratumumab, lenalidomide and dexamethasone	NDMM
			MagnetisMM-7 (NCT05317416) [95]	Monotherapy	NDMM
			MagnestisMM-8 (NCT05228470) [96]	Monotherapy	RRMM
			MagnestisMM-9 (NCT05014412) [97]	Monotherapy	RRMM
REGN5458	BCMA x CD3	Phase I/II	LINKER-MM1 (NCT03761108) [98]	Monotherapy	RRMM
			NCT05137054 [98]	Combination with anti-cancer therapies	RRMM
ABBV-383	BCMA x CD3	Phase I/II	NCT03933735 [20]	Monotherapy	RRMM
Pavurutamab	BCMA x CD3	Phase I	ParadigMM-1B (NCT03287908) [99]	Monotherapy and combination with pomalidomide with/without dexamethasone	RRMM
Alnuctamab	BCMA x CD3	Phase I	NCT03486067 [34]	Monotherapy	RRMM
WVT078	BCMA x CD3	Phase I	NCT04123418 [100]	Monotherapy and in combination with WHG626	MM
Talquetamab*	GPRC5D x CD3	Phase III	MonumenTAL-1 (NCT03399799) [30]	Monotherapy	RRMM
			MonumenTAL-2 (NCT05050097) [101]	Combination with anticancer therapies	RRMM
			MonumenTAL-3 (NCT05455320) [102]	Combination with daratumumab with/without pomalidomide	RRMM
			MonumenTAL-5 (NCT05461209) [103]	Monotherapy	RRMM
			NCT04654552 [104]	Monotherapy	RRMM
			NCT04773522 [105]	Monotherapy	RRMM
RG6234	GPRC5D x CD3	Phase I	NCT04557150 [35]	Monotherapy	RRMM
Cevostamab	FcRH5 x CD3	Phase I/II	CAMMA 1 (NCT04910568) [106]	Monotherapy and combination with pomalidomide/daratumumab and dexamethasone	RRMM
			CAMMA 2 (NCT05535244) [107]	Monotherapy	BCMA-exposed RRMM
			PLYCOM (NCT05583617) [108]	Combination with tocilizumab and lenalidomide	MM
			NCT03275103 [10]	Monotherapy	RRMM
ISB 1342	CD38 x CD3	Phase I	NCT03309111 [109]	Monotherapy	Previously treated MM

Note, this table does not include expanded access programs, compassionate use, or retrospective studies.

*Refer to teclistamab section for studies investigating talquetamab and teclistamab combination therapies.

Patients with MM that become refractory to the three major MM treatment classes: immunomodulatory drugs (IMiDs), PIs, and anti-CD38 monoclonal antibodies (mAbs), are referred to as ‘triple-class refractory’ [11]. The prognosis of triple-class refractory MM patients is poor [12], and treatment options for these patients are limited. With currently available treatments, patients have a median overall survival of 12.4 months (95% confidence interval [CI], 10.3–NE) [13].

Novel immune therapies are now becoming available for RRMM, including CAR T-cell therapies idecabtagene vicleucel (ide-cel) and ciltacabtagene autoleucel (cilta-cel), as well as BsAbs [14, 15]. The toxicities associated with BCMA-targeted CAR T-cell therapies and BCMA-targeted BsAbs are similar, due to the similarities in their immune mechanisms of action, although frequencies and severity may vary between both modalities [16, 17].

An increased infection risk has been observed with BsAbs, as compared with conventional MM treatment regimens [18, 19]. The spectrum of toxicity associated with BsAbs includes adverse events (AEs), such as cytokine release syndrome (CRS) [20], immune effector cell-associated neurotoxicity syndrome [21], and peripheral neuropathy [22], as well as AEs which contribute to increased infection risk, such as cytopenias and hypogammaglobulinemia (HGG), and infection AEs themselves [23, 24]. In addition, a propensity for opportunistic infections associated with defects in T cell and/or B cell immunity has been observed in clinical trials with BsAbs for MM [25], which can lead to increased risk of serious conditions such as *pneumocystis jirovecii* pneumonia (PJP), and reactivation of cytomegalovirus (CMV) and hepatitis B virus (HBV) [26–28]. With BCMA-targeting BsAbs, infection rates range between 33% and 76%, indicating an increased infection risk through BCMA signaling effects [22, 24].

In addition, MM, a disease of the immune system, is frequently associated with significant immune impairment and dysfunction of the adaptive immune response, due to malfunction of the immune regulation of plasma cells [29]. This results in an increased risk of infections in this population [30].

Currently, there are general guidelines and recommendations available for the management and prophylaxis of infections in patients with MM, as well as CAR T-cell guidelines for infection management [16, 17, 31]. As BsAbs become a treatment option for MM, guidance on the diagnosis of infections, monitoring, prophylaxis, and treatment is needed.

This review summarizes the discussions of a panel of global experts and provides consensus recommendations based on available clinical evidence and clinical experience, which inform on infection risk, prophylaxis, and management of patients receiving BsAb monotherapy or combination therapy.

## Methodology

A panel of 13 experts from Europe and North America developed the recommendations herein; this panel comprised 12 MM experts and one infectious disease expert.

A systematic literature review was performed in PubMed and across all abstracts from relevant congresses, ASCO, EHA, and ASH, from 1st January 2019 until 19th July 2022, to identify relevant information on infections with all BsAbs in patients with MM, using the search terms of “multiple myeloma”, “infection”, and “bispecific antibodies”. Specific terms can be found in Table 2. Primary articles that were published in English were assessed for relevancy, to ensure inclusion of all papers and abstracts with clinical data with BsAbs. For clinical trials with multiple data cutoffs, the most recent data were used. Following the literature review, updated trial data have been added where relevant and reviewed by the panel.

**Table 2:** Search terms for systematic literature review conducted 1st January 2019 until 19th July 2022.

General search terms*	BsAb search terms*	Infection search terms*
• Multiple myeloma • Infection • Bispecific antibodies	• Teclistamab • Elranatamab • REGN5458 • Pavurutamab • ABBV-383 • Pacanalotamab • Alnuctamab • WVT078 • Talquetamab • RG6234 • Cevostamab • ISB 1342	• *Pneumocystis pneumonia* • *Pneumocystis jirovecii* • Respiratory infection • Progressive multifocal leukoencephalopathy • Cytomegalovirus • Hepatitis • Viral reactivation • Bacterial infection • Fungal infection • COVID-19

^*^Search terms included derivatives of these terms and variations in the nomenclature of therapeutic agents.

Seven experts convened at the International Myeloma Workshop in Los Angeles, CA on 24th August 2022 to review the literature results. In addition, a survey was constructed prior to the workshop for discussion at the meeting to gain insight and ascertain the level of agreement regarding the panel’s recommendations for the treatment and management of these patients. The survey was sent out in advance of the meeting and completed by three additional experts who were unable to attend in person.

Consensus recommendations were reached through live and offline discussions. The panel used grading criteria post-meeting to assess the level of recommendations:

- Level I: empirical; however, requires significantly more data to support it
- Level IIA and IIB: empirical, with slightly more data available to support the recommendation
- Level IIC: based on routine practice, with sufficient supporting evidence
- Level III: considered to be obligatory practice, with strong available evidence.

All 13 authors contributed in full to discussions following the meeting and survey, in order to develop the recommendations in this report. Each author provided their recommendations on a Scale of 1–5 (corresponding with Levels I–III, respectively, as detailed above), and the average scores were calculated and rounded to the nearest integer.

## Literature search results overview

### Infection data across clinical trials in MM patients with BsAb

As many BsAbs are still in the early stages of development, currently available infection data are limited. Clinical trials with teclistamab and elranatamab included large sample sizes, with 165 patients in the teclistamab MajesTEC-1 study and 123 patients in Cohort A (no prior BCMA-directed treatment) of the elranatamab MagnetisMM-3 study (Table 3) [24, 32].

**Table 3:** Literature summary of infection data from BsAb clinical trials in MM patients.

Drug	Target	Study	Phase	Safety cohort *N*	No. of patients receiving RP2D *n*	Duration of treatment (range)	Infection AEs *n* (%)	Treatment-related infection AEs *n* (%)	Serious infection TEAEs *n* (%)	Infection AEs leading to discontinuation *n* (%)	Infection AEs resulting in death *n* (%)	Neutropenia *n* (%)	Lymphopenia *n* (%)	Leukopenia *n* (%)
Teclistamab		MajesTEC-1 [24]	I/II	165^a^	165	8.5 months (0.2–24.4)	Any grade: (76.4)	Any grade: –	–	2 (1.21)^b†^	16 (8.48%)^c^	Any grade: 117^d^ (70.9)	Any grade: 57 (34.5%)	Any grade: 29 (17.6)
							Grade 3/4: (44.8)	Grade 3/4: –			Considered related: 4 (2.4)	Grade 3/4: 106 (64.2)	Grade 3/4: 54 (32.7)	Grade 3/4: 12 (7.3)
	BCMA x CD3	MajesTEC-2 [40] (+daratumumab +lenalidomide)	Ib	32^e^	19	–	Any grade: 29 (90.6)	–	–	2 (6.3)^f^	2 (6.3)^g^	Any grade: 27 (84.4)	Any grade: 4 (12.5)	–
							Grade 3/4: 12 (37.5)					Grade 3/4: 25 (78.1)	Grade 3/4: 4 (12.5)	
Elranatamab	BCMA x CD3	MagnetisMM-1 [46–48]	I	55^h^	–	–	–	–	–	–	1	Any grade: 41 (74.5)^h^	Any grade: 29 (52.7)^h^	Any grade: 19 (34.5)^i^
							Grade 3/4: 15 (27.3)					Grade 3/4: 39 (71.0)^h^	Grade 3/4: 28 (51.0)^h^	Grade 3/4: 13 (23.7)^i^
		MagnetisMM-3 [32]	II	123^j^	123	5.6 months (0.03–19.8)	Any grade: (66.7)	–	–	8 (6.5)	2 (1.6)	Any grade: 59 (48.0)	Any grade: 32 (26.0)	–
							Grade 3/4: (35.0)					Grade 3/4: 59 (48.0)	Grade 3/4: 30 (24.4%)	
REGN5458	BCMA x CD3	LINKER-MM1 [110–113]	I/II	68^k^	–	–	Any grade: (46.7)^l^	–	1 (14.3%)^m^	–	5 (6.85)^k^	Any grade: 17 (23)^n^	Any grade: 17 (23)^n^	–
							Grade 3/4: (20.0)^l^				Considered related: 0^k^	Grade 3/4: 16 (22)^n^	Grade 3/4: 14 (19.2)^n^	–
Pavurutamab	BCMA x CD3	NCT03287908 [114]	I/II	75^o^	–	6.1 weeks (3.1–15.3)	–	–	13 (17%)	1	2 (2.67)	Any grade: 23%	–	–
											Considered related: 0 (0)	Grade 3/4: –	–	–
ABBV-383	BCMA x CD3	NCT03933735 [20, 49]	I/II	174^p^	–	–	Grade ≥3: 39 (22)	–	–	–	4 (3.2)^q^	Any grade: 60 (34)	Any grade: 45 (26)	–
											Considered related: 0 (0)	Grade 3/4: 45 (26)	Grade 3/4: 41 (24)	–
Pacanalotamab	BCMA x CD3	NCT02514239 [22]	I	42	10	–	Any grade: 14 (33)	–	14 (33.3)	–	2 (4.7)^r^	–	–	–
							Grade 3–5: 10 (24.0)							
Alnuctamab	BCMA x CD3	NCT03486067 [34]	I	–	–	14.0 weeks (1.6–46.0)	Grade 3/4: 30.0%^s^	–	1^s^	–	–	Grade 3/4: 43%^s^	–	–
WVT078	BCMA x CD3	NCT04123418 [100]	I	33^t^	–	–	–	–	–	–	–	Grade ≥3: 12.1%	Grade ≥3: 18.2%	–
Talquetamab	GPRC5D x CD3	MonumenTAL-1 [9, 33, 37, 115]	I/II	0.4 mg/kg QW: 143^u^	288	–	Any grade: 46.7%^v^	–	–	–	–	Any grade: 49 (34.3)	Any grade: 40 (28.0)	Any grade: 12 (40.0)^v^
							Grade 3/4: 6.7%^v^					Grade 3/4: 44 (30.8)	Grade 3/4: 37 (25.9)	Grade 3/4: 9 (30.0)^v^
				0.8 mg/kg Q2W: 145^u^			Any grade: 38.6%^v^	–		–	–	Any grade: 41 (28.3)	Any grade: 38 (26.2)	Any grade: 10 (22.7)^v^
							Grade 3/4: 9.1%^v^					Grade 3/4: 32 (22.1)	Grade 3/4: 37 (25.5)	Grade 3/4: 8 (18.2)^t^
RG6234	GPRC5D x CD3	NCT04557150 [35]	I	–	–	–	Any grade: 27 (52.9)^w^	–	–	2	2	Any grade: 12 (23.6)^w^	–	–
							Grade 3/4: 9 (17.6)^w^					Grade 3/4: 6 (11.8)^w^		
Cevostamab	FcRH5 x CD3	NCT03275103 [10]	I	160^x^	–	–	Any grade: 68 (42.5)	–	–	–	–	Any grade: 29 (18.10)	–	–
							Grade 3/4: 30 (18.8)	–	–	–	–	Grade 3/4: 26 (16.3)	–	–
Talquetamab + Daratumumab	GPRC5D x CD3	TRIMM-2 [39]	Ib	58 ^y^	–	–	Any grade: 31 (53.4)		–	–	1 (1.72)^z†^	Any grade: 19 (32.8)	Any grade: 15 (25.9)	–
							Grade 3/4: 10 (17.2)		–	–	Considered related: 1 (1.72)	Grade 3/4: 13 (22.4)	Grade 3/4: 15 (25.9)	
Teclistamab + Daratumumab	BCMA x CD3	TRIMM-2 [38]	Ib	65^z^	–	–	Any grade: 44 (67.7)	–	–	–	3 (4.62)^aa^	Any grade: 32 (49.2)	–	–
							Grade 3/4: 18 (27.7)		–	–	Considered related: 0	Grade 3/4: 27 (41.5)	–	–

Cells with no data are where data was not provided for the variable.

^a^Data cutoff: 16 March 2022, median follow-up: 14.1 months. ^b^1 due to Grade 3 adenoviral pneumonia, 1 due to Grade 4 PML. ^c^12 due to COVID-19, 1 due to streptococcal pneumonia, 1 due to PML, 1 due to pneumonia, 1 due to pseudomonal pneumonia. ^d^1 patient had a dose reduction due to recurrent neutropenia. 91 patients received G-CSF for neutropenia. ^e^Data cutoff: 17 Oct 2022. ^f^Both due to COVID-19. ^g^One due to COVID-19, one due to multi-organ failure due to sepsis. ^h^Data cutoff: 30 September 2022. Median follow-up: 12.0 months. ^i^Data cutoff: 26 July 2021. Median follow-up: 12.5 months (part 1), 7.5 months (part 1.1). ^j^Data cutoff: October 14, 2022. Median follow-up: 10.4 months. ^k^Data cutoff: 10 June 2021, median follow-up: 2.4 months. ^l^Interim analysis, *N* = 45, Data cutoff: 15 June 2020, median follow-up: 2.37 months. ^m^Interim analysis, *N* = 7, Data cutoff: 11 Oct 2019, follow-up range: 1.8–7.5 months. ^n^Unknown data cutoff, safety population *n* = 73. ^o^Data cutoff: 2 July 2020, median follow-up: 1.7 months. ^p^Data cutoff: 16 Aug 2022, median follow-up: 14.1 months. ^q^Data cutoff: 8 Jan 2022, median follow-up: 10.8 months. In this data cutoff: three died due to COVID-19, one due to sepsis. ^r^One due to influenza/ aspergillosis, and one due to adenovirus-related hepatitis. ^s^Data cutoff: 28 October 2019. Median follow-up not provided. ^t^Data cutoff: 8 December 2021. Median follow-up not provided. ^u^Data cutoff 12 September 2022, median follow-up for 0.4 mg/kg: 14.9 months, for 0.8 mg/kg: 8.6 months. ^v^Data cutoff 6 Apr 2022, median follow-up for 0.4 mg/kg: 13.2 months, for 0.8 mg/kg: 7.7 months. ^w^Data cutoff: 5 Apr 2022. Median follow-up not provided. ^x^Data cutoff: 18 May 2021, median follow-up: 6.1 months. ^y^Data cutoff 6 April 2022, talquetamab median follow-up: 5.1 months, teclistamab median follow-up: 8.6 months. ^z^Due to treatment-related pneumonia. ^aa^Due to treatment-related bacterial pneumonia, sepsis, COVID-19, considered unrelated to treatment.

*AE* adverse event, *BCMA* B cell maturation agent, *BsAb* bispecific antibody, *CD* cluster of differentiation, *FcRH5* Fc receptor-homolog 5 *G-CSF* granulocyte colony-stimulating factor, *MM* multiple myeloma, *PML* progressive multifocal leukoencephalopathy, *Q2W* once every 2 weeks, *RP2D* recommended Phase 2 dose, *R/R* relapsed/refractory, *TEAE* treatment-emergent adverse event.

In the Phase I/II teclistamab MajesTEC-1 study, 76.4% of patients experienced any grade infection AEs, 44.8% experienced Grade 3/4 infection-related AEs. Median follow-up was 14.1 months (0.3–24.4) and median duration of treatment (DoT) was 8.5 months (0.2–24.4). The most common infection AEs in the MajesTEC-1 trial were pneumonia and COVID-19, experienced by 18.2% and 17.6% of patients, respectively. Two patients discontinued treatment due to infection AEs of Grade 3 adenoviral pneumonia and Grade 4 progressive multifocal leukoencephalopathy. Of those who experienced infection AEs, 16 patients (9.7%) died, due to COVID-19 (12 patients), pneumonia (one patient), pseudomonal pneumonia (one patient), streptococcal pneumonia (one patient) and progressive multifocal leukoencephalopathy (one patient) [24].

In the Phase II elranatamab MagnetisMM-3 study, among 123 patients without history of prior BCMA-directed therapy, 66.7% experienced any-grade infection AEs [32], 35.0% Grade 3/4. The median follow-up was 10.4 months, and median DoT was 5.6 months (0.03–19.8) [32]. The most common infection AEs were COVID-19-related AEs and upper respiratory tract infection, occurring in 25.2% and 17.9% of patients, respectively. Eight patients discontinued treatment due to infection-related AEs, most commonly septic shock (*n* = 2) and sepsis (*n* = 2). PJP was reported in 6 (4.9%) patients (5 [4.1%] Grade 3/4), CMV reactivation in 6 (4.9%) patients (2 [1.6%] Grade 3/4), and CMV infection in 4 (3.3%) patients (no Grade 3/4). One patient died due to pseudomonal pneumonia considered related to elranatamab by investigator; Grade 5 infection AEs unrelated to elranatamab occurred in five patients (4.1%), including two cases of COVID-19 pneumonia [32].

In the MonumenTAL-1 study with talquetamab, rates of Grade 3/4 infections were observed to be lower than the rates reported with elranatamab or teclistamab: 57.3% of patients receiving the 0.4 mg/kg dose experienced any grade infections, 16.8% Grade 3/4. Of the patients receiving the 0.8 mg/kg dose, 50.3% experienced any grade infections, 11.7% Grade 3/4 [33].

In clinical trials with other BsAbs, infection AE incidence varied between 32.0% and 52.9%, with 6.7% to 30.0% ≥Grade 3 events (Table 3) [34–37]. These trials are limited, due to the small sample sizes, inclusion of different dose cohorts, and short follow-up duration, and thus should be followed for more mature data.

Incidence of infection with combination of teclistamab or talquetamab with daratumumab in the small Phase I/II TRIMM-2 study was consistent with monotherapy data [38, 39]. 53.4% of patients experienced any grade infection and 17.2% experienced Grade 3/4 infections with talquetamab combination, 67.7% and 27.7% experienced any grade and Grade 3/4 infections, respectively, with teclistamab combination [38, 39]. However, in the MajesTEC-2 trial, in which teclistamab was administered in combination with daratumumab and lenalidomide, 90.6% and 37.5% of patients experienced any grade and Grade 3/4 infections, respectively [40].

## Consensus recommendations

It was recognized by the expert panel members that the data for each individual BsAb monotherapy and combination therapy are not mature enough to draw from for each recommendation at this stage, as many BsAbs are currently in early development, and data have not yet been published.

The experts were unanimous in their decision to base recommendations for all BsAb monotherapies and combinations largely on data from the teclistamab Phase I/II MajestTEC-1 study and the elranatamab Phase II MagnetisMM-3 study, with some focus on the smaller clinical trials with other BsAbs, as well as drawing from their own clinical experience. This was due to these trials having the largest patient populations receiving BsAbs at the Phase II recommended dose. It should be noted that of the smaller BsAb studies, different doses were evaluated in subgroups, and therefore the reported incidence of infections is based on differently dosed patient groups.

The expert panel members recognize that BsAbs with targets other than BCMA, such as GPRC5D and Fc receptor-homolog 5 (FcRH5), may have variable infection rates and risks, depending on doses, dose intervals, and patient characteristics, and future recommendations may need to take into account these variables. It should be considered that the current data from BsAb clinical trials were conducted in a heavily pre-treated patient population, so are likely to have an increased risk of infections. Table 4 provides a summary of the key recommendations in this document.

**Table 4:** Overview of management, treatment, and prophylaxis recommendations for patients with MM receiving BsAbs.

		Monitoring	Prophylaxis	Treatment	Action with BsAb
Risk factors					
HGG		• Pay particular attention to Ig levels in this patient population (level IIC) • IgG and IgM serology tests for diagnosis of viral infections may be used routinely, but interpreted with caution (level IIC) • Monitor Ig levels monthly during Ig treatment (level IIC)	–	• The expert panel discussed Ig replacement therapy for the following patients: ◦ Patients whose IgG levels <400 mg/dl (level IIC) ◦ Patients who have ≥2 severe recurrent infections by encapsulated bacteria, regardless of IgG level (level IIC) ◦ Patients with a life-threatening infection (level III) ◦ Patients with documented bacterial infection with no or insufficient response to antibiotic therapy (level IIC) • Monthly IVIG treatment recommended for the duration of immunoparesis, and in absence of life-threatening infectious manifestations, until Ig levels are ≥400 mg/dl (level IIC)	• Maintain dosing during Ig treatment (level IIC)
Neutropenia		–	• Anti-bacterial or anti-fungal prophylaxis should be considered if neutropenia is prolonged or chronic despite G-CSF treatment (level IIC)	• Colony-stimulating factors recommended in patients with documented ≥Grade 3 neutropenia (level III) • Avoid G-CSF during periods when a patient is at risk of CRS (level IIB)	• Withhold dosing in neutropenia cases where absolute neutrophil count <0.5 × 10^9^/L, or in febrile neutropenia cases, until neutrophil count has returned to normal levels (level IIB)
Infections					
Viral infection	General	• Based on patient symptoms and clinical presentation (level IIC) • PCR-based viral panels to diagnose viral infections and reactivations (level III)	• Acyclovir or valacyclovir against HSV and VZV in all RRMM patients (level III) • Monitoring is not recommended while using these prophylactic treatments (IIB) • Prophylaxis should be maintained whilst the patient is still receiving treatment for MM, and thereafter at the discretion of the individual physician (level III)	• Dependent on infectious agent	• Maintain dosing during prophylaxis • Temporary discontinuation during anti-viral treatment until clinical resolution of infectious symptoms, or until the viral load is not clinically significant (level IIC)
	CMV	• If infection risk is suspected, monitor using CMV DNA copies (level IIC)	–	• Oral valganciclovir for CMV reactivation (level IIC) • Alternatives: IV ganciclovir of foscarnet (level IIC) • Follow standard guidelines for CMV reactivation	–
	EBV	• Not routinely recommended, but should be considered (level IIC) • In cases of persistent fever and fatigue, monitor EBV DNA copies in order to exclude EBV DNA reactivation (level IIB)	–	• Rituximab in post-allogenic HSCT patients (level IIA)	–
	VZV	–	• Acyclovir or valacyclovir (level III) • Vaccination against VZV recommended in RRMM patients (level IIB)	• Valacyclovir or IV acyclovir for VZV reactivation (as per standard treatment guidelines), different agents may be used if following local guidelines (level III)	–
	HBV	• Screen for core antibodies (level III) and surface antigens prior to starting MM treatment (level III) • Monitor HBV DNA copies in core antigen positive patients (level III)	• If core antibody positive, administer prophylaxis, or monitor for HBV DNA copies, with pre-emptive anti-viral treatment for those with positive DNA tests/viremia (level III) • If surface antigen positive, administer anti-viral prophylaxis: entecavir, tenofovir, lamivudine under the control of specialists, as per standard treatment guidelines (level III)	–	• Maintain dosing during prophylaxis (level III) • Discontinue if patient experiences reactivation (level IIC)
	Influenza	• Direct testing of nasopharyngeal or respiratory secretions by PCR if influenza is suspected (level III)	• Influenza vaccination of patients receiving BsAbs and close contacts recommended (level III) • Two-dose series, at least one month apart, of high-dose influenza vaccine may increase likelihood of seroprotection (level IIC)	• Oseltamivir or baloxivir, if influenza is confirmed, as per standard guidelines (level IIC)	–
	SARS-COV-2	• Follow center protocols (level III) • If COVID-19 is suspected, PCR test on nasal, nasopharyngeal or respiratory secretions (level III)	• Follow CDC guidelines or local health authority guidelines for vaccination (level III) • Treat with mAbs with proven efficacy against the prevalent variant (if effective prophylactic antibodies are available) (level IIC)	• Treat with available therapies, with consideration of concurrent medications; treatment is based on symptoms and physician assessment (level III)	• Temporary discontinuation in patients with COVID-19 until clinical resolution, together with RT-PCR clearance (level III)
Bacterial infection		• Blood, urine, sputum and fecal cultures. Test choice depends on infection site (level III) • Imaging to provide greater insight and confirm extent of infection (level III) • For further confirmation: imaging such as CT or PET-CT scans for pneumonia evaluation, suspected colitis, diverticulitis or abdominal abscesses, or procedural biopsy based on the infection site (level III)	• Recommended in patients with: ◦ Prolonged neutropenia (level IIC) ◦ High risk of infections (level IIC) ◦ History of recurrent bacterial infections (level IIC) • In these circumstances, treat with levofloxacin, stopping treatment once patient no longer has neutropenia (level IIC) • Risk of developing resistant pathogens should be considered with use of anti-bacterial prophylaxis (level IIC) • Combining anti-bacterial prophylactic treatments is not recommended (level IIC)	• Dependent on infectious agent, targeted therapy recommended if agent can be identified (level III) ◦ Broad-spectrum antibiotics for patients with concomitant neutropenia (level III) ◦ Levofloxacin or equivalent, based on site of infection, for patients who do not have concomitant neutropenia (level IIC) ◦ For older patients who those with QT prolongation: third generation cephalosporins (level IIC) • Treat until symptoms resolve (level III) • Treating microbial colonizations is not recommended (level IIC), however treatment may be used in very immunocompromised patients (level IIB)	• Maintain dosing during prophylaxis (level III) • Temporary discontinuation, until infection resolution, during anti-bacterial treatment (level III)
Fungal infection	General	• Routine monitoring is not recommended (level IIC) • Serum galactomannan testing if aspergillosis is suspected (level IIC) • Cultures, imaging, and diagnostic tests help identify the fungal infection, if suspected (level III) • Biopsy to confirm mold in patient with sinusitis (level III)	• Not recommended (level IIC), unless patient has previous history of fungal infections (level IIC), prolonged neutropenia (level IIC), or history of prolonged high-dose corticosteroid use (<2 weeks) (IIC) • Administer prophylaxis following consultation with an infectious disease specialist, if available (level III) • If using prophylaxis: fluconazole is recommended (level IIC) • Itraconazole and voriconazole can be considered (level IIC) • Monitoring during anti-fungal prophylaxis is not recommended, unless for suspected aspergillosis, and depending on patient risk (level IIC)	• Dependent on infectious agent and investigations • Treat as per infectious disease guidelines, and consult with an infectious disease provider (level III)	• Maintain dosing during prophylaxis, when needed (level III) • Temporarily discontinue during anti-fungal treatment, until symptom resolution (level III)
	*P. jirovecii* infection	• Routine monitoring is not recommended (level III) • Review each case individually (level III)	• Recommended prophylaxis for all patients (level III) • Trimethoprim-sulfamethoxazole (level IIC), dapsone or atovaquone if allergic to sulfonamide (level IIC) • Inhaled or intravenous pentamidine for patients with neutropenia (level IIB)	• Treat as per standard anti-microbial regimens for PJP: ◦ Trimethoprim-sulfamethoxazole for 21 days (level III) ◦ Atovaquone 750 mg po BID for 21 days (for mild cases, sulfonamide allergy) (level III) ◦ Clindamycin and primaquine for 21 days (for moderate/severe cases, sulfonamide allergy) (level IIC)	• Maintain dosing during prophylaxis (level IIC)
Vaccinations					
–		–	• Follow general guidelines on use of live attenuated vaccines, however it should be noted that live vaccines are contraindicated in MM patients (level IIC) • Ensure post-stem cell transplant vaccinations have been carried out (level III) • COVID-19 vaccination recommended as per CDC guidelines, in patients without history of transplant (level III) • Yearly influenza vaccination, pneumococcal vaccination and varicella zoster vaccination recommended in patients without history of transplant (level III) • Close contacts should receive seasonal vaccines (level III) • Health-care providers caring for MM patients should be fully immunized and should receive seasonal vaccines (level III) • Prior to traveling to endemic areas of infection, patients should receive travel vaccinations and undergo consultation with a disease specialist (level III)	–	–

*BID* twice daily, *BsAb* bispecific antibody, *CD* cluster of differentiation, *CDC* Center for Disease Control, *CMV* cytomegalovirus, *COVID-19* coronavirus 19, *CT* computerized tomography, *EBV* Epstein-Barr virus, *HBV* hepatitis B virus, *HSCT* hematopoietic stem cell transplantation, *HSV* herpes simplex virus, *MM* multiple myeloma, *PCR* polymerase chain reaction, *PET-CT* positron emission tomography CT, *PJP* *pneumocystis jirovecii* pneumonia, *RRMM* relapsed refractory multiple myeloma, *SARS-COV-2* syndrome coronavirus 2, *VZV* varicella zoster virus.

### Risk factors for infection

#### Risk factors overview

**Fig. 1:**
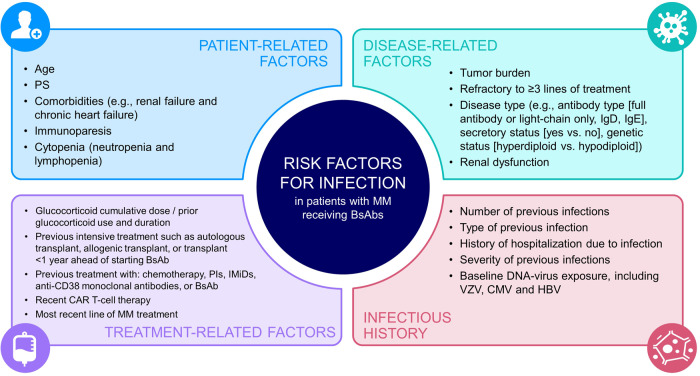
Risk factors for infection in patients with MM receiving BsAbs. BsAb bispecific antibody, CAR-T chimeric antigen receptor T-cell, CMV cytomegalovirus, HBV hepatitis B virus, IMiD immunomodulatory drug, MM multiple myeloma, PI proteasome inhibitors, PS propensity score, VZV varicella zoster virus.

Noting that all MM patients receiving BsAbs warrant a high degree of vigilance for infection risk and occurrence, the panel categorized risk factors, from the BsAb literature reviewed, by patient-, disease-, and treatment-related factors (Fig. 1).

Some of the risk factors warrant consideration for specific prophylactic approaches prior to initiation of BsAb treatment, based on data from other therapeutic classes.

#### Hypogammaglobulinemia (HGG)

##### Background

MM patients commonly experience secondary immune deficiencies such as HGG [23], a disorder defined by low serum IgG levels [41]. HGG increases infection risk with encapsulated bacteria, and is associated with decreased overall survival [23]. It has been observed that treatment with BsAbs can lead to prolonged HGG, which in turn is associated with increased infection risk [18].

##### Recommendations for the management of hypogammaglobulinemia

*Monitoring*

As hypogammaglobulinemia is frequent in this patient population, particular attention to immunoglobulin (Ig) levels is recommended (level IIC). IgG and IgM serology tests for the diagnosis of past viral infections may be used routinely, but interpreted with caution in this population, as patients have often received intravenous immunoglobin (IVIG) treatment, which may impact the results (level IIC). Patients may also have a false negative result in response to IgG and IgM serology tests, due to failure to mount antibody responses to pathogens. A false negative IgG test can make it harder to reliably understand important past exposures in the population, such as CMV. Therefore it is important to test serological status at baseline. However, caution should be taken with baseline serology tests if a patient is negative for IgG CMV.

*Treatment*

The expert panel discussed Ig replacement therapy for the following patients:

- Patients whose IgG levels <400 mg/dl (level IIC)
- Patients who have experienced ≥2 severe recurrent infections by encapsulated bacteria, regardless of IgG level (level IIC)
- Patients with a life-threatening infection (level III)
- Patients with documented bacterial infection with no or insufficient response to antibiotic therapy (level IIC)

The panel recommend monthly IVIG treatment for the duration of immunoparesis, and in the absence of life-threatening infectious manifestations, until Ig levels are ≥400 mg/dl (level IIC). Ig levels should be monitored monthly during Ig treatment (level IIC). It is important to note that serum levels alone are not adequate to inform on an individual’s capacity to mount an antibody response against various pathogens, and it is more important to monitor the frequency of infections (level IIC). We recommend maintaining BsAb dosing during Ig treatment (level IIC).

##### Literature summary

Data are very limited regarding the incidence of treatment-emergent HGG with BsAbs. In the MajesTEC-1 trial, 123 (74.5%) of patients in the safety population (*N* = 165) experienced treatment-emergent HGG, as determined by AE reporting, laboratory results (IgG <500 mg/dl), or both. Of these patients, more than half (*n* = 65) received IVIG at the physician’s discretion [24]. In the MagnetisMM-3 study, among patients with available laboratory results, 76 patients (75.2%) had IgG levels <400 mg/dl. 50 patients (40.7%) received IVIG during the study [32]. With talquetamab in the MonumenTAL-1 trial, HGG occurred in 87% of patients who had received treatment with 0.4 mg/kg, and in 71% who had received 0.8 mg/kg [9]. In a Phase I, first-in-human study with ABBV-383, 14% of patients in the trial (*N* = 124) reported treatment-emergent HGG AEs, while 23% required immunoglobulin administration [20].

#### Neutropenia

##### Background

MM patients are at risk of developing neutropenia, which can result in increased risk of serious infections and febrile neutropenia [42, 43]. Neutropenia-related infections may be potentially life-threatening, resulting in treatment delays or dose modification, which in turn can reduce treatment efficacy [42]. Neutropenia can be treated by using recombinant granulocyte colony stimulating factor (G-CSF) [43]. Appropriate use of G-CSF prophylaxis is important to reduce the risk of infection in MM patients [43].

##### Recommendations for the management of neutropenia

We recommend use of colony-stimulating factors in patients with documented Grade ≥3 neutropenia (level III). If neutropenia is prolonged or chronic despite G-CSF treatment, anti-bacterial or anti-fungal prophylaxis should be considered (level IIC) (see section below for general recommendations on anti-bacterial prophylaxis). G-CSF use should be avoided during periods when a patient is at risk of CRS [44], due to the overlapping symptomology of febrile neutropenia and CRS, and the release of cytokines with G-CSF treatment (level IIB) [44]. In line with the FDA prescribing information for teclistamab, it is recommended to withhold BsAb dosing in neutropenia cases where absolute neutrophil count <0.5 ×10^9^/L or in febrile neutropenia cases [45], until neutrophil count has returned to normal levels.

##### Literature summary

Incidence of neutropenia has been recorded across a number of clinical trials with BsAbs in MM patients [46]. In the Phase I MagnetisMM-1 study with elranatamab, 74.5% experienced any grade, and 71.0% experienced Grade 3/4 neutropenia [47, 48]. In the Phase II MagnetisMM-3 study, 48.0% experienced Grade 3/4 neutropenia [32]. With teclistamab, 64.2% experienced Grade 3/4 neutropenia in Phase I/II MajesTEC-1 study. In this study, one patient had a BsAb dose reduction due to recurrent neutropenia, and 91 (55.1% patients) received treatment with G-CSF [24]. In the Phase I study with ABBV-383, 34% experienced any grade, and 26% experienced Grade 3 neutropenia [20, 49].

### Monitoring for suspected infections

#### Recommendations for the identification of suspected infections

Laboratory tests, metabolic panels, and imaging should remain as standard clinical practice, and should be carried out based on symptoms and clinical presentation, in order to identify the infection. Specific recommendations from the panel for viral, bacterial, and fungal infections are provided in the following sections of the manuscript and can be found in Table 4.

### Viral infections

#### Background

RRMM patients are at high risk of life-threatening reactivation of viral infections, such as herpes simplex virus (HSV), varicella zoster virus (VZV), and HBV [17, 50], and acquisition of new viral infections such as influenza or severe acute respiratory syndrome coronavirus 2 (SARS-CoV-2), which can lead to severe complications and death [17, 51].

RRMM patients may have increased risk of CMV reactivation, which is associated with increased morbidity and mortality, and can result in severe end-organ disease [52, 53]. The incidence of CMV reactivation in MM patients receiving BsAbs is uncertain, due to limited clinical trial experience to-date [52, 53].

MM patients with SARS-CoV-2 infection have an increased risk of severe outcomes [31, 54–56]. Risk is likely increased with BsAb treatment, due to depletion of functional plasma cells [57]. While the relationship between BsAb treatment and rate of severe disease due to SARS-CoV-2 infection is unknown, it has been observed that patients receiving myeloma treatment, including BsAbs, are less likely to develop antibodies in response to SARS-CoV-2 vaccination [58]. Use of a booster dose can significantly improve antibody levels in MM patients; however, patients treated with anti-BCMA agents are less likely to achieve neutralizing antibody levels above the positivity threshold [56].

#### Recommendations for prophylaxis and management of viral infections

##### Monitoring

Monitoring should be carried out based on symptoms and clinical presentation. The use of PCR-based viral panels is recommended to diagnose active viral infections and reactivations (level IIC).

##### Prophylaxis

Acyclovir or valacyclovir are recommended as anti-viral prophylaxis against HSV and VZV in all RRMM patients (level III). Prophylaxis should be maintained whilst the patient is still receiving treatment for MM, and thereafter at the discretion of the individual physician. Monitoring while using these prophylactic treatments is not recommended by the expert panel (level IIB).

##### Treatment of emergent viral infections

Recommendations on anti-viral treatment type and duration are dependent on the infectious agent. Prior to anti-viral treatments, documentation of the viral infection through clinical manifestations, physical examinations, and anti-microbial tests is recommended (level IIC). In line with the FDA Prescribing Information (PI) for teclistamab, we recommend temporary discontinuation of BsAbs during anti-viral treatment until clinical resolution of infection symptoms or until the viral load is not clinically significant (level IIC) [45].

##### Cytomegalovirus (CMV)

We recommend collection of baseline CMV history and IgG and IgM status prior to BsAb treatment, independent of suspected infection (level IIC). If CMV infection risk is suspected, baseline quantification and monitoring of CMV DNA copies are recommended (level IIC).

We recommend treating CMV reactivation with oral valganciclovir (level IIC) or alternatively with intravenous ganciclovir or foscarnet, as per standard guidelines for CMV reactivation (level IIC) [17]. Valganciclovir may be used to treat patients with preserved bone marrow function (level IIC). One expert also considers letermovir an option for prophylaxis based on evidence from patients receiving allogeneic transplant [59].

##### Epstein-Barr virus (EBV)

Monitoring EBV DNA copies is not routinely recommended but should be considered. In cases of persistent fever and fatigue, monitoring EBV DNA copies may be considered for the possibility of EBV reactivation (level IIC) [60].

Treatment options for EBV include rituximab, which has also shown promise as prophylaxis against EBV reactivation in post-allogenic hematopoietic stem cell transplantation (HSCT) [61].

##### Varicella zoster virus

The panel recommends anti-viral prophylaxis (treatments described in the earlier viral prophylaxis section) (level III). It is recommended the VZV reactivation is treated with valacyclovir or IV acyclovir (level III), as per standard treatment guidelines; [17] however, different agents may be used if following local guidelines (level III). We recommend that RRMM patients are vaccinated against VZV (level IIC); there are currently no clear data on stopping anti-viral prophylaxis following vaccination.

##### Hepatitis B virus

We recommend HBV screening for core antibodies (level III) and surface antigens (level III) prior to starting treatment in MM patients. For patients who are core antibody positive, we recommend either administering prophylaxis, or monitoring for HBV DNA copies (level III), with pre-emptive anti-viral treatment for those with positive DNA tests/viremia (level III). Patients who are surface antigen positive and/or have positive HBV DNA should receive anti-viral therapy and be treated with entecavir, tenofovir, or lamivudine under the control of specialists, as per standard treatment guidelines [17].

We recommend maintaining BsAb dosing during prophylactic anti-hepatitis treatment (level III); however, as per the FDA PI for teclistamab, teclistamab should be discontinued if a patient experiences reactivation [45].

##### Influenza virus

Direct influenza testing of nasopharyngeal or respiratory secretions by PCR is recommended for suspected influenza (level III). If influenza is confirmed, treatment with oseltamivir or baloxivir is recommended, as per standard treatment guidelines (level IIC) [17].

Influenza vaccination of patients receiving BsAbs and close contacts (e.g., household members and caregivers) is recommended (level III). Use of a two-dose series, at least one month apart, of high-dose influenza vaccine may increase likelihood of seroprotection (level IIC) [62].

##### SARS-COV-2

It is recognized that COVID-19 monitoring requirements may vary between centers. We, therefore, recommend following center protocols for monitoring. If COVID-19 is suspected, a PCR test on nasal, nasopharyngeal, or respiratory secretions is recommended to confirm diagnosis (level III).

For COVID-19 vaccination, we recommend following Centers for Disease Control and Prevention (CDC) or local health authority guidelines [63].

As per routine clinical practice, we recommend treatment with the available therapies of all COVID-19-positive patients based on symptoms and physician assessment, considering the patient’s concurrent medications, and being aware of drug-drug interactions. For prophylaxis, we recommend treatment with monoclonal antibodies with proven efficacy against the prevalent variant, if available. We recommended that BsAb treatment is temporarily discontinued in patients with COVID-19 (level III) until clinical resolution, together with RT-PCR clearance testing. If the patient is asymptomatic, a high cycle threshold (Ct) may be used to measure resolution. It should be noted that PCR testing can remain positive for a long time period, in these cases rapid antigen testing can also be used to confirm resolution. If the patient continues to have positive COVID-19 testing, consider consultation with an infectious disease specialist.

#### Literature summary

Viral infections and reactivations have been reported across a number of clinical trials with BsAbs in MM patients (Table 3). It should be noted that patients who were HBV core antibody positive were excluded from clinical trials with BsAbs, so risk of HBV reactivation is currently unknown.

### Bacterial infections

#### Background

MM patients are seven times more susceptible to bacterial infections than age- and sex-matched controls, with an 11-fold higher risk during the first year following diagnosis [17, 50]. Antibiotic prophylaxis has been demonstrated to decrease the overall incidence of infection in MM patients within the first three months following diagnosis [64].

#### Recommendations for prophylaxis and management of bacterial infections

##### Monitoring

To confirm the bacterial agent, as per standard clinical practice, we recommend identification using blood, urine, sputum, and fecal cultures; choice of test/culture source depends on infection site. Imaging can also provide greater insight into the areas and extent of bacterial infection [65, 66]. If further confirmation is required, use imaging, such as CT or PET-CT scans for pneumonia evaluation, suspected colitis, diverticulitis or abdominal abscesses, or procedural biopsy based on the infection site.

##### Prophylaxis

General anti-bacterial prophylaxis is not recommended in patients receiving BsAbs (level III). However, we recommend use of anti-bacterial prophylaxis for patients with prolonged neutropenia (level IIC). Anti-bacterial prophylaxis is also recommended in patients who are at high risk of infections and patients with a history of recurrent bacterial infections (level IIC). If using anti-bacterial prophylaxis, we recommend levofloxacin, stopping treatment once the risk is lower (i.e., the patient no longer has neutropenia) (level IIC).

In line with standard clinical practice, the risk of development of resistant pathogens should be considered with use of anti-bacterial prophylaxis, although data from a large study did not confirm an increased risk for development of resistant strains [67].

We do not recommend combining anti-bacterial prophylactic treatments, but do recommend maintaining BsAb dosing during anti-bacterial prophylaxis (level IIC).

##### Treatment of emergent bacterial infections

If the infectious agent can be identified, we recommend targeted therapy, as per standard clinical practice.

Broad-spectrum antibiotics are recommended for patients with concomitant neutropenia (level III). Levofloxacin or equivalent, based on site of infection, is recommended for the treatment of patients without concomitant neutropenia (level IIC). For older patients, or those with QT prolongation, we recommend third-generation cephalosporins (level IIC). Treatment of bacterial infections is recommended until symptoms resolve.

We do not recommend treating microbial colonizations (level III). However, treatment may be used in very immunocompromised patients (level IIB).

We recommend temporarily discontinuing BsAbs during active anti-bacterial treatment until infection resolution (level III).

#### Literature summary

While bacterial infection incidence while receiving BsAbs has been scarcely captured in clinical trials, we have provided the recommendations based on our own clinical experience.

### Fungal infections

#### Background

Fungal infections in MM patients who have undergone HSCT, or who are receiving immunosuppressive immune therapies, may be associated with early mortality [68]. PJP is a rare opportunistic fungal infection that can result in life-threatening pneumonia in patients with hematological malignancies such as MM [69]. The mortality rate of PJP in these patients is 30–60% [69].

Although PJP is rare in patients with MM [17], use of immunomodulatory therapies can result in patients being at risk of infection, with PJP being diagnosed in patients who would typically be considered lower risk [70]. Early recognition of at-risk patients and the disease is critical for optimal management [69].

#### Recommendations for prophylaxis and management of fungal infections

##### Monitoring

Routine fungal testing with β-glucan or galactomannan tests is not recommended (level IIC). It is recognized that β-glucan tests can often be falsely positive in this patient population, due to patients receiving IVIG treatment. If aspergillosis is suspected, we recommend serum galactomannan testing (level IIC). Cultures, imaging, and diagnostic tests can help identify the fungal infection, if suspected (level III). If imaging is concerning for a patient with sinusitis, it is recommended to consult an ear, nose, and throat specialist to perform a biopsy, confirming fungal infections (level III).

##### Prophylaxis

With the exception for *P. jirovecii* (see below), anti-fungal prophylaxis is not recommended, unless a patient has a previous history of fungal infections (level IIC), prolonged neutropenia (level IIC), or has recently received prolonged treatment with high dose corticosteroids (>2 weeks) (level IIC). If, following consultation with an infectious disease specialist, prophylaxis is needed, fluconazole is recommended (level IIC). Itraconazole and voriconazole may also be considered (level IIC). Monitoring during anti-fungal prophylaxis is not recommended, unless for suspected aspergillosis (level IIC). We recommend maintaining BsAb treatment during anti-fungal prophylaxis, when needed (level III).

##### Treatment of emergent fungal infections

Patients with invasive fungal infections, such as invasive candidiasis or Aspergillosis should be treated per standard infectious diseases guidelines [71], preferably in consultation with an infectious diseases provider. We recommend temporarily discontinuing BsAb treatment during anti-fungal treatment, until symptom resolution (level III).

#### *Pneumocystis jirovecii*

Routine monitoring for *P. jirovecii* infection is not recommended (level III); however, due to the high mortality of PJP, and due to the prevalence of PJP in teclistamab and elranatamab clinical trials (3.6–4.9%), we suggest that each case is individually reviewed (level III). Anti-PJP prophylaxis is recommended for all patients (level III). Trimethoprim-sulfamethoxazole, dapsone, or atovaquone is recommended for prophylaxis (level IIC), with the latter two options for patients allergic to sulfonamide. For patients with neutropenia, prophylaxis with intravenous or inhaled pentamidine are alternatives to dapsone, trimethoprim-sulfamethoxazole, or atovaquone (level IIB). We recommend maintaining BsAb dosing during anti-PJP prophylaxis (level III).

#### Literature summary

In the Phase I/II MajesTEC-1 teclistamab trial, no patients experienced Grade ≥3 fungal infections, but six patients (3.6%) developed serious PJP pneumonia. Eight patients (5%) received anti-fungal prophylaxis [24, 72]. In the Phase II MagnetisMM-3 elranatamab trial, 4.9% and 4.1% of patients experienced any grade and Grade 3/4 PJP, respectively [32].

### Vaccinations

#### Background

Appropriate use of vaccinations is important in MM patients, to produce immune responses and prevent potentially harmful infections. However, MM patients often have a lower response to vaccinations, so additional guidance may be required [73].

#### Vaccine recommendations for patients receiving BsAbs

We recommend following general guidelines on the use of live attenuated vaccines (level IIC) [31, 73]; however, it should be noted that live vaccines are contraindicated in MM patients, with the exception of those with complete immune reconstitution (patients who underwent autologous stem cell transplant [ASCT] >24 months prior). Immunity following vaccination is not guaranteed in these patients, as immune response is variable across vaccine recipients, and is particularly unpredictable among patients with MM due to the immune-regulating treatments they receive. It has been observed that vaccinations in patients with MM may induce protective T-cell responses even in the absence of antibody responses [74].

It must be ensured that post-stem cell transplant vaccinations have been carried out (level III). In patients without a history of transplant, it is recommended that patients receive COVID-19 vaccination as per CDC guidelines (level III), yearly influenza vaccination (level III), pneumococcal vaccine (level III), and the varicella zoster vaccine (level III).

To reduce the infection risk, it is recommended that close contacts receive seasonal vaccines (level III). We cannot recommend avoiding close contact with recipients of live vaccines, due to the difficulty of achieving this (level IIC). However, it is recommended that healthcare providers caring for these patients should be fully immunized and should receive seasonal influenza vaccines (level III). Prior to traveling to endemic areas of infection, patients should receive travel vaccinations and undergo consultation with an infectious disease specialist (level III).

#### Literature summary

There are currently no clinical trial data regarding the use of vaccines in patients receiving BsAbs.

## Discussion

This review summarizes the recommendations of a global expert panel and can be used to inform management of infection risks, including prophylaxis and treatment, and guide supportive measures against risk factors for MM patients receiving BsAb monotherapy or combination therapy.

Parallels can be drawn between the recommendations for infection monitoring and prophylaxis with BsAbs and recommendations with CAR T-cell therapies [16]. It is recognized that there is more real-world experience with CAR T-cell therapies, and learnings can be drawn from this population regarding infection risk, prophylaxis, and treatment, when treating with BsAbs [16, 75, 76]. While there are many similarities in the guidance for patients receiving treatment with BsAbs and CAR T-cell therapy, it should be noted that there are minor differences, and BsAbs have their own individual infection risks.

While the expert panel considered making specific recommendations for each individual and BsAb combination therapy, it was recognized that at this current time the available data are limited. Thus, the expert panel was unanimous in providing recommendations for all BsAb monotherapies and combinations. As the clinical BsAb data evolves, recommendations will develop based on BsAb target and disease stage. As more data emerge, it will be of interest to observe any differences in infection risk between BsAbs according to target antigen, and challenges with combination regimens comprising BsAbs and standard-of-care agents with overlapping toxicity profiles. Studies in which BsAbs are used for an extensive period of time, as maintenance treatment, will reveal the long-term infection profile, which will be important to evaluate and aid development of long-term management guidelines.

When interpreting these recommendations, clinicians should consider that each recommendation is based on clinical evidence, as well as clinical experience and knowledge gained through daily practice, in addition to local/institutional guidelines, as well as national and international guidelines for the treatment of MM patients. Although the recommendations herein are intended as a guide to assist with timely and informed decisions, they should not replace sound clinical judgment or be used as a legal resource. It is essential that physicians and patients consult an infectious disease expert for guidance when appropriate and where possible, regarding diagnosis and management of infections.

Further specific data from clinical trials would be useful to reinforce opinions and support recommendations, these data include type of infection and mortality rate, patient’s history of prior infections (i.e., baseline viral, bacterial, and fungal infection history), prior therapies, timing (early vs. late in treatment), infection duration, and prophylaxis offered.

It should be acknowledged that real-world data are often very different from clinical trial data. More real-world data will emerge as BsAbs are used more frequently in clinical practice and will inform our understanding in this area.

## Conclusion

This current expert consensus provides graded recommendations for MM patients receiving BsAb treatment. Additional expert panel meetings will be required in the future, following the emergence of new data, to determine any necessary recommendation updates, and potentially provide specific recommendations for individual BsAb monotherapies and combinations.

## Data sharing

Data for preparation of this manuscript, in the form of the survey results, was shared with all authors, in accordance with Pfizer’s data sharing policy.

## References

[1] Moreau P, Touzeau C (2022). T-cell–redirecting bispecific antibodies in multiple myeloma: a revolution?. Blood.

[2] Chng WJ (2022). New immunotherapeutic target in myeloma. Blood.

[3] Lancman G, Sastow DL, Cho HJ, Jagannath S, Madduri D, Parekh SS (2021). Bispecific antibodies in multiple myeloma: present and future. Blood Cancer Discov.

[4] Xu H, Cheng M, Guo H, Chen Y, Huse M, Cheung NK (2015). Retargeting T cells to GD2 pentasaccharide on human tumors using Bispecific humanized antibody. Cancer Immunol Res.

[5] EMA. New medicine for multiple myeloma patients with limited treatment options. 2022. https://www.ema.europa.eu/en/news/new-medicine-multiple-myeloma-patients-limited-treatment-options.

[6] U.S. FDA. FDA approves teclistamab-cqyv for relapsed or refractory multiple myeloma. 2022. https://www.fda.gov/drugs/resources-information-approved-drugs/fda-approves-teclistamab-cqyv-relapsed-or-refractory-multiple-myeloma.

[7] Pfizer. Pfizer’s elranatamab granted FDA breakthrough therapy designation for relapsed or refractory multiple myeloma. 2022. https://www.pfizer.com/news/press-release/press-release-detail/pfizers-elranatamab-granted-fda-breakthrough-therapy.

[8] Pfizer. Pfizer’s elranatamab receives FDA and EMA filing acceptance. 2023. https://www.pfizer.com/news/press-release/press-release-detail/pfizers-elranatamab-receives-fda-and-ema-filing-acceptance.

[9] Chari A, Minnema MC, Berdeja JG, Oriol A, van de Donk NWCJ, Rodríguez-Otero P (2022). Talquetamab, a T-cell–redirecting GPRC5D bispecific antibody for multiple myeloma. N Engl J Med.

[10] Sebag M, Raje NS, Bahlis NJ, Costello C, Dholaria B, Solh M (2021). Elranatamab (PF-06863135), a B-cell maturation antigen (BCMA) targeted CD3-engaging bispecific molecule, for patients with relapsed or refractory multiple myeloma: results from magnetismm-1. Blood.

[11] Costa LJ, Hungria V, Mohty M, Mateos MV (2022). How I treat triple-class refractory multiple myeloma. Br J Haematol.

[12] Gandhi UH, Cornell RF, Lakshman A, Gahvari ZJ, McGehee E, Jagosky MH (2019). Outcomes of patients with multiple myeloma refractory to CD38-targeted monoclonal antibody therapy. Leukemia.

[13] Mateos M-V, Weisel K, De Stefano V, Goldschmidt H, Delforge M, Mohty M (2022). LocoMMotion: a prospective, non-interventional, multinational study of real-life current standards of care in patients with relapsed and/or refractory multiple myeloma. Leukemia.

[14] U.S. FDA. FDA approves first cell-based gene therapy for adult patients with multiple myeloma. 2021. https://www.fda.gov/news-events/press-announcements/fda-approves-first-cell-based-gene-therapy-adult-patients-multiple-myeloma.

[15] U.S. FDA. FDA approves ciltacabtagene autoleucel for relapsed or refractory multiple myeloma. 2022. https://www.fda.gov/drugs/resources-information-approved-drugs/fda-approves-ciltacabtagene-autoleucel-relapsed-or-refractory-multiple-myeloma.

[16] Los-Arcos I, Iacoboni G, Aguilar-Guisado M, Alsina-Manrique L, Díaz de Heredia C, Fortuny-Guasch C (2021). Recommendations for screening, monitoring, prevention, and prophylaxis of infections in adult and pediatric patients receiving CAR T-cell therapy: a position paper. Infection.

[17] Raje NS, Anaissie E, Kumar SK, Lonial S, Martin T, Gertz MA (2022). Consensus guidelines and recommendations for infection prevention in multiple myeloma: a report from the International Myeloma Working Group. Lancet Haematol.

[18] Hammons LR, Szabo A, Janardan A, Dhakal B, Chhabra S, D’Souza A (2022). Kinetics of humoral immunodeficiency with bispecific antibody therapy in relapsed refractory multiple myeloma. JAMA Netw Open.

[19] Saez A, Lopez-Muñoz N, Sánchez-Pina JM, Alonso R, cuellar C, lázaro P (2022). P-113: infectious toxicities in patients treated with bispecific antibodies in multiple myeloma. Clin Lymphoma Myeloma Leuk.

[20] D’Souza, A, Shah, N, Rodriguez, C, Voorhees, PM, Weisel, K, Bueno, OF et al. A Phase I First-in-Human Study of ABBV-383, a B-cell maturation antigen × CD3 bispecific T-cell redirecting antibody, in patients with relapsed/refractory multiple myeloma. J Clin Oncol. 2022;40:3576–86. 10.1200/JCO.22.01504 PMC9622641 36029527

[21] Subklewe M (2021). BiTEs better than CAR T cells. Blood Adv.

[22] Topp MS, Duell J, Zugmaier G, Attal M, Moreau P, Langer C (2020). Anti-B-cell maturation antigen BiTE molecule AMG 420 induces responses in multiple myeloma. J Clin Oncol.

[23] Lancman G, Lozada K, Athar N, Jacobs S, Doucette J, Cho HJ (2021). Efficacy of intravenous immunoglobulin for preventing infections in patients with multiple myeloma. Clin Lymphoma Myeloma Leuk.

[24] Moreau P, Garfall AL, van de Donk N, Nahi H, San-Miguel JF, Oriol A (2022). Teclistamab in relapsed or refractory multiple myeloma. N Engl J Med.

[25] Mohan M, Nagavally S, Dhakal B, Radhakrishnan SV, Chhabra S, D’Souza A (2022). Risk of infections with B-cell maturation antigen-directed immunotherapy in multiple myeloma. Blood Adv.

[26] Khanam A, Chua JV, Kottilil S (2021). Immunopathology of chronic hepatitis B infection: role of innate and adaptive immune response in disease progression. Int J Mol Sci.

[27] Kolls JK (2017). An emerging role of B cell immunity in susceptibility to pneumocystis pneumonia. Am J Respir Cell Mol Biol.

[28] Pera A, Vasudev A, Tan C, Kared H, Solana R, Larbi A (2017). CMV induces expansion of highly polyfunctional CD4+ T cell subset coexpressing CD57 and CD154. J Leukoc Biol.

[29] Leblay, N, Maity, R, Hasan, F & Neri, P Deregulation of adaptive T cell immunity in multiple myeloma: insights into mechanisms and therapeutic opportunities. Front Oncol. 2020;10:636. 10.3389/fonc.2020.00636 PMC7214816 32432039

[30] Caro J, Braunstein M, Williams L, Bruno B, Kaminetzky D, Siegel A (2022). Inflammation and infection in plasma cell disorders: how pathogens shape the fate of patients. Leukemia.

[31] Ludwig, H & Kumar, S. Prevention of infections including vaccination strategies in multiple myeloma. Am J Hematol. 2022;98:S46–62. 10.1002/ajh.26766 36251367

[32] Bahlis, NJ, Tomasson, MH, Mohty, M, Niesvizky, R, Nooka, AK, Manier, S et al. Efficacy and safety of elranatamab in patients with relapsed/refractory multiple myeloma naïve to B-cell maturation antigen (BCMA)-directed therapies: results from cohort a of the magnetismm-3 study. 64th American Society of Hematology (ASH) Annual Meeting and Exposition, 2022.

[33] Chari, A e. a. Talquetamab, a G protein-coupled receptor family c group 5 member D x CD3 bispecific antibody, in patients with relapsed/refractory multiple myeloma: phase 1/2 results from monumenTAL-1. American Society of Hematology (ASH) Annual Meeting and Exposition, 2022.

[34] Costa, LJ. Interim results from the first phase 1 clinical study of the b-cell maturation antigen (BCMA) 2+1 T cell engager (TCE) CC-93269 in patients (PTS) with relapsed/refractory multiple myeloma (RRMM). European Hematology Association (EHA) Abstract: S205 (2020).

[35] Hasselbalch Riley C, Hutchings M, Yoon S-S, Koh Y, Manier S, Facon T (2022). S180: RG6234, a novel GPRC5D T-cell engaging bispecific antibody, induces rapid responses in patients with relapsed/refractory multiple myeloma: preliminary results from a first-in-human trial. Hemasphere.

[36] Kumar S, D’Souza A, Shah N, Rodriguez C, Voorhees P, Bueno O (2021). A phase 1 first-in-human study of Tnb-383B, a BCMA x CD3 bispecific T-cell redirecting antibody, in patients with relapsed/refractory multiple myeloma. Blood.

[37] Minnema, M. Efficacy and safety of talquetamab, a G protein-coupled receptor family C group 5 member D x CD3 bispecific antibody, in patients with relapsed/refractory multiple myeloma: updated results from monumenTAL-1. Am Soc Clin Oncol, 2022;40:8015.

[38] Rodriguez-Otero, P, D’Souza, A, Reece, DE, Van de Donk, N, Chari, A, Krishnan, A. et al. Teclistamab in combination with daratumumab, a novel, immunotherapy-based approach for the treatment of relapsed/refractory multiple myeloma: updated phase 1b results. European Hematology Association (EHA) Abstract no. S188, 2022.

[39] van de Donk, N, Bahlis, N, Mateos, MV, Weisel, K, Dholaria, B, Garfall, AL et al. Novel combination immunotherapy for the treatment of relapse/refractory multiple myeloma: updated phase 1b results for talquetamab (A GPRC5D x CD3 bispecific antibody) in combination with daratumumab. European Hematology Association (EHA) Abstract no. S183, 2022.

[40] Searle, E, Quach, H, Wong, SH, Megala Costa, LJ, Hulin, C, Janowski, W et al. Teclistamab in combination with subcutaneous daratumumab and lenalidomide in patients with multiple myeloma: results from one cohort of MajesTEC-2, a Phase1b, multicohort study. 64th American Society of Hematology (ASH) Annual Meeting and Exposition, 2022.

[41] Huq M, BN. Hostoffer RW in StatPearls [Internet]. StatPearls Publishing, 2022.

[42] Ding X, Ding J, Gu H, Zhong C (2022). Long-acting granulocyte colony-stimulating factor in primary prophylaxis of early infection in patients with newly diagnosed multiple myeloma. Support Care Cancer.

[43] Leleu X, Gay F, Flament A, Allcott K, Delforge M (2018). Incidence of neutropenia and use of granulocyte colony-stimulating factors in multiple myeloma: is current clinical practice adequate. Ann Hematol.

[44] Lee DW, Gardner R, Porter DL, Louis CU, Ahmed N, Jensen M (2014). Current concepts in the diagnosis and management of cytokine release syndrome. Blood.

[45] TECVAYLI® (teclistamab-cqyv) Prescribing Information. 2022. https://www.accessdata.fda.gov/drugsatfda_docs/label/2022/761291s000lbl.pdf.

[46] Jakubowiak AJ, Bahlis NJ, Raje NS, Costello C, Dholaria BR, Solh MM (2022). Elranatamab, a BCMA-targeted T-cell redirecting immunotherapy, for patients with relapsed or refractory multiple myeloma: updated results from MagnetisMM-1. J Clin Oncol.

[47] Raje, N, Bahlis, NJ, Costello, C, Dholaria, B, Solh, M, Levy, MY et al. Elranatamab, a BCMA targeted T-cell engaging bispecific antibody, induces durable clinical and molecular responses for patients with relapsed or refractory multiple myeloma. 64th American Society of Hematology (ASH) Annual Meeting and Exposition, 2022.

[48] Sebag, M. MagnetisMM-1: a study of elranatamab, a B-cell maturation antigen (BCMA)-targeted CD3-engaging bispecific molecule, for patients with relapsed or refractory multiple myeloma. ASH, 2021.

[49] Voorhees, P. A Phase 1 first-in-human study of ABBV-383, a BCMA x CD3 bispecific T-Cell-redirecting Antibody, As Monotherapy In Patients With Relapsed/refractory Multiple Myeloma. American Society Of Hematology (ASH) Annual Meeting and Exposition, 2022. 10.1200/JCO.22.01504 PMC9622641 36029527

[50] Blimark C, Holmberg E, Mellqvist UH, Landgren O, Björkholm M, Hultcrantz M (2015). Multiple myeloma and infections: a population-based study on 9253 multiple myeloma patients. Haematologica.

[51] Encinas C, Hernandez-Rivas J-Á, Oriol A, Rosiñol L, Blanchard M-J, Bellón J-M (2022). A simple score to predict early severe infections in patients with newly diagnosed multiple myeloma. Blood Cancer J.

[52] Hasegawa T, Aisa Y, Shimazaki K, Ito C, Nakazato T (2016). Cytomegalovirus reactivation in patients with multiple myeloma. Eur J Haematol.

[53] Tay, KH, Slavin, MA, Thursky, KA, Coussement, J, Worth, LJ, Teh, BW et al. Cytomegalovirus DNAemia and disease: current-era epidemiology, clinical characteristics and outcomes in cancer patients other than allogeneic haemopoietic transplantation. Int Med J. 2022;52:1759–67. 10.1111/imj.15496 34448333

[54] Chari A, Samur MK, Martinez-Lopez J, Cook G, Biran N, Yong K (2020). Clinical features associated with COVID-19 outcome in multiple myeloma: first results from the International Myeloma Society data set. Blood.

[55] Ludwig H, Sonneveld P, Facon T, San-Miguel J, Avet-Loiseau H, Mohty M (2021). COVID-19 vaccination in patients with multiple myeloma: a consensus of the European Myeloma Network. Lancet Haematol.

[56] Terpos E (2022). Vaccination against SARS-CoV-2 for myeloma patients: do we need a booster dose and how frequent?. Hematol Transfus Cell Ther.

[57] Dilillo DJ, Olson K, Mohrs K, Meagher TC, Bray K, Sineshchekova O (2018). REGN5458, a bispecific BCMAxCD3 T cell engaging antibody, demonstrates robust in vitro and in vivo anti-tumor efficacy in multiple myeloma models, comparable to that of BCMA CAR T Cells. Blood.

[58] Van Oekelen O, Gleason CR, Agte S, Srivastava K, Beach KF, Aleman A (2021). Highly variable SARS-CoV-2 spike antibody responses to two doses of COVID-19 RNA vaccination in patients with multiple myeloma. Cancer Cell.

[59] Marty FM, Ljungman P, Chemaly RF, Maertens J, Dadwal SS, Duarte RF (2017). Letermovir prophylaxis for cytomegalovirus in hematopoietic-cell transplantation. N Engl J Med.

[60] Clave E, Agbalika F, Bajzik V, Peffault de Latour R, Trillard M, Rabian C (2004). Epstein-barr virus (EBV) reactivation in allogeneic stem-cell transplantation: relationship between viral load, EBV-specific T-cell reconstitution and rituximab therapy. Transplantation.

[61] Wei N, Wang Y, Wang J, Wu L, Wang Z (2021). Characteristics of Epstein-Barr virus reactivation after allogeneic haematopoietic stem cell transplantation in patients with chronic active Epstein-Barr virus disease: favorable responses to rituximab. Bone Marrow Transplant.

[62] Branagan AR, Duffy E, Albrecht RA, Cooper DL, Seropian S, Parker TL (2017). Clinical and serologic responses after a two-dose series of high-dose influenza vaccine in plasma cell disorders: a prospective, single-arm trial. Clin Lymphoma Myeloma Leuk.

[63] (CDC), C. f. D. C. a. P. Stay up to date with COVID-19 vaccines including boosters, https://www.cdc.gov/coronavirus/2019-ncov/vaccines/stay-up-to-date.html (2023).

[64] Mohyuddin GR, Aziz M, McClune B, Abdallah AO, Qazilbash M (2020). Antibiotic prophylaxis for patients with newly diagnosed multiple myeloma: systematic review and meta-analysis. Eur J Hematol.

[65] Ordonez, AA, Sellmyer, MA, Gowrishankar, G, Ruiz-Bedoya, CA, Tucker, EW, Palestro, CJ et al. Molecular imaging of bacterial infections: overcoming the barriers to clinical translation. Sci Transl Med. 2019;11:eaax8251. 10.1126/scitranslmed.aax8251 PMC6743081 31484790

[66] Polvoy I, Flavell RR, Rosenberg OS, Ohliger MA, Wilson DM (2020). Nuclear imaging of bacterial infection: the state of the art and future directions. J Nucl Med.

[67] Drayson, M, Bowcock, S, Planche, T, Iqbal, G, Pratt, G, Yong, K et al. Levofloxacin prophylaxis in patients with newly diagnosed myeloma (TEAMM): a multicentre, double-blind, placebo-controlled, randomised, phase 3 trial. Lancet Oncol. 2019;20:1760–72. 10.1016/S1470-2045(19)30506-6 PMC6891230 31668592

[68] Tsai C-K, Liu Y-C, Kuan AS, Lee K-L, Yeh C-M, Lee Y-T (2020). Risk and impact of invasive fungal infections in patients with multiple myeloma. Ann Hematol.

[69] Cordonnier C, Cesaro S, Maschmeyer G, Einsele H, Donnelly JP, Alanio A (2016). Pneumocystis jirovecii pneumonia: still a concern in patients with haematological malignancies and stem cell transplant recipients. J Antimicrob Chemother.

[70] White PL, Price JS, Backx M (2018). Therapy and management of pneumocystis jirovecii Infection. J Fungi.

[71] Pappas PG, Kauffman CA, Andes DR, Clancy CJ, Marr KA, Ostrosky-Zeichner L (2015). Clinical practice guideline for the management of candidiasis: 2016 update by the Infectious Diseases Society of America. Clin Infect. Dis.

[72] Usmani SZ, Garfall AL, van de Donk NWCJ, Nahi H, San-Miguel JF, Oriol A (2021). Teclistamab, a B-cell maturation antigen x CD3 bispecific antibody, in patients with relapsed or refractory multiple myeloma (MajesTEC-1): a multicentre, open-label, single-arm, phase 1 study. Lancet.

[73] Ludwig H, Boccadoro M, Moreau P, San-Miguel J, Cavo M, Pawlyn C (2021). Recommendations for vaccination in multiple myeloma: a consensus of the european myeloma network. Leukemia.

[74] Aleman A, Upadhyaya B, Tuballes K, Kappes K, Gleason CR, Beach K (2021). Variable cellular responses to SARS-CoV-2 in fully vaccinated patients with multiple myeloma. Cancer Cell.

[75] Brioli A, Klaus M, Sayer H, Scholl S, Ernst T, Hilgendorf I (2019). The risk of infections in multiple myeloma before and after the advent of novel agents: a 12-year survey. Ann Hematol.

[76] Mikkilineni L, Kochenderfer JN (2021). CAR T cell therapies for patients with multiple myeloma. Nat Rev Clin Oncol.

[77] ClinicalTrials.gov. A study of teclistamab with other anticancer therapies in participants with multiple myeloma (MajesTEC-2), (2022).

[78] ClinicalTrials.gov. A study of teclistamab in combination with daratumumab subcutaneously (SC) (Tec-Dara) versus daratumumab sc, pomalidomide, and dexamethasone (DPd) or daratumumab SC, bortezomib, and dexamethasone (DVd) in participants with relapsed or refractory multiple myeloma (MajesTEC-3). 2022. https://clinicaltrials.gov/ct2/show/NCT05083169.

[79] ClinicalTrials.gov. A study of teclistamab in combination with lenalidomide versus lenalidomide alone in participants with newly diagnosed multiple myeloma as maintenance therapy following autologous stem cell transplantation (MajesTEC-4). 2022. https://clinicaltrials.gov/ct2/show/NCT05243797.

[80] ClinicalTrials.gov. A study to compare teclistamab in combination with daratumumab and lenalidomide (Tec-DR) in participants with newly diagnosed multiple myeloma (MajesTEC-7). 2022. https://clinicaltrials.gov/ct2/show/NCT05552222.

[81] ClinicalTrials.gov. A study comparing teclistamab monotherapy versus pomalidomide, bortezomib, dexamethasone (PVd) or carfilzomib, dexamethasone (Kd) in participants with relapsed or refractory multiple myeloma (MajesTEC-9). 2022, https://clinicaltrials.gov/ct2/show/NCT05572515.

[82] ClinicalTrials.gov. A study of teclistamab in japanese participants with relapsed or refractory multiple myeloma. 2022. https://clinicaltrials.gov/ct2/show/NCT04696809.

[83] ClinicalTrials.gov. Study of teclistamab in combination in elderly patients with multiple myeloma (IFM 2021-01). 2022. https://clinicaltrials.gov/ct2/show/NCT5572229.

[84] ClinicalTrials.gov. A study of the combination of talquetamab and teclistamab in participants with relapsed or refractory multiple myeloma (RedirecTT-1). 2022. https://clinicaltrials.gov/ct2/show/NCT04586426.

[85] ClinicalTrials.gov. A study of talquetamab and teclistamab each in combination with a programmed cell death receptor-1 (pd-1) inhibitor for the treatment of participants with relapsed or refractory multiple myeloma (TRIMM-3). 2022. https://clinicaltrials.gov/ct2/show/NCT05338775.

[86] ClinicalTrials.gov. Immuno-PRISM (PRecision Intervention Smoldering Myeloma). 2022. https://clinicaltrials.gov/ct2/show/NCT05469893.

[87] ClinicalTrials.gov. Sequential therapy in multiple myeloma guided by MRD assessments (MASTER-2). 2022. https://clinicaltrials.gov/ct2/show/NCT05231629.

[88] ClinicalTrials.gov. A study of subcutaneous daratumumab regimens in combination with bispecific t cell redirection antibodies for the treatment of participants with multiple myeloma. 2022. https://clinicaltrials.gov/ct2/show/NCT04108195.

[89] ClinicalTrials.gov. PF-06863135 as single agent and in combination with immunomodulatory agents in relapse/refractory multiple myeloma. 2022. https://clinicaltrials.gov/ct2/show/NCT03269136.

[90] ClinicalTrials.gov. MAGNETISMM-2: study of elranatamab (PF-06863135) in Japanese participants with multiple myeloma. 2022. https://clinicaltrials.gov/ct2/show/NCT04798586.

[91] ClinicalTrials.gov. MagnetisMM-3: study of elranatamab (PF-06863135) monotherapy in participants with multiple myeloma who are refractory to at least one PI, one IMiD and one anti-CD38 mAb. 2022. https://clinicaltrials.gov/ct2/show/NCT04649359.

[92] ClinicalTrials.gov. MagnetisMM-4: umbrella study of elranatamab (PF-06863135) in combination with anti-cancer treatments in multiple myeloma. 2022. https://clinicaltrials.gov/ct2/show/NCT05090566.

[93] ClinicalTrials.gov. MagnetisMM-5: study of elranatamab (PF-06863135) monotherapy and elranatamab + daratumumab versus daratumumab + pomalidomide + dexamethasone in participants with relapsed/refractory multiple myeloma (MAGNETISMM-5). 2022. https://clinicaltrials.gov/ct2/show/NCT05020236.

[94] ClinicalTrials.gov. A study to learn about the effects of the combination of elranatamab (PF-06863135), daratumumab, and lenalidomide compared with daratumumab, lenalidomide, and dexamethasone in patients with newly diagnosed multiple myeloma who are not candidates for transplant (MagnetisMM-6). 2022. https://www.clinicaltrials.gov/ct2/show/NCT05623020.

[95] ClinicalTrials.gov. Study with elranatamab versus lenalidomide in patients with newly diagnosed multiple myeloma after transplant (MagnetisMM-7). 2022. https://clinicaltrials.gov/ct2/show/NCT05317416.

[96] ClinicalTrials.gov. MagnetisMM-8: Study of elranatamab (PF-06863135) monotherapy in chinese participants with refractory multiple myeloma. 2022. https://clinicaltrials.gov/ct2/show/NCT05228470.

[97] ClinicalTrials.gov. A study to learn about the study medicine (elranatamab) in participants with multiple myeloma that has come back after responding to treatment or has not responded to treatment (MagnetisMM-9). 2022. https://clinicaltrials.gov/ct2/show/NCT05014412.

[98] ClinicalTrials.gov. REGN5458 (Anti-BCMA x Anti-CD3 bispecific antibody) plus other cancer treatments for participants with relapsed/refractory multiple myeloma. 2022. https://clinicaltrials.gov/ct2/show/NCT05137054

[99] ClinicalTrials.gov. A study to assess AMG 701 montherapy, or in combination with pomalidomide, with or without, dexamethasone in subjects with relapsed or refractory multiple myeloma. 2022. https://clinicaltrials.gov/ct2/show/NCT03287908.

[100] Raab MS, Cohen YC, Schjesvold F, Aardalen K, Oka A, Spencer A (2022). P937: preclinical discovery and early findings from the phase 1, dose-escalation study of wvt078, a Bcma-cD3 bispecific antibody, in patients with R/R multiple myeloma. Hemasphere.

[101] ClinicalTrials.gov. A study of talquetamab with other anticancer therapies in participants with multiple myeloma (MonumenTAL-2). 2022. https://clinicaltrials.gov/ct2/show/NCT05050097.

[102] ClinicalTrials.gov. A study comparing talquetamab in combination with daratumumab or in combination with daratumumab and pomalidomide versus daratumumab in combination with pomalidomide and dexamethasone in participants with relapsed or refractory multiple myeloma (MonumenTAL-3). 2022. https://clinicaltrials.gov/ct2/show/NCT05455320.

[103] ClinicalTrials.gov. A study of comparing talquetamab to belantamab mafodotin in participants with relapsed/refractory multiple myeloma (MonumenTAL-5). 2022. https://clinicaltrials.gov/ct2/show/NCT05461209.

[104] ClinicalTrials.gov. A study of talquetamab in participants with relapsed or refractory multiple myeloma. 2022. https://clinicaltrials.gov/ct2/show/NCT04634552.

[105] ClinicalTrials.gov. A study of JNJ-64407564 in Japanese participants with relapsed or refractory multiple myeloma. 2022. https://clinicaltrials.gov/ct2/show/NCT04773522.

[106] ClinicalTrials.gov. A study evaluating the safety, pharmacokinetics, and activity of cevostamab in participants with relapsed or refractory multiple myeloma (CAMMA 1). 2022. https://clinicaltrials.gov/ct2/show/NCT04910568.

[107] ClinicalTrials.gov. A study evaluating the efficacy and safety of cevostamab in prior b cell maturation antigen (bcma)-exposed participants with relapsed/refractory multiple myeloma (CAMMA 2). 2022. https://clinicaltrials.gov/ct2/show/NCT05535244.

[108] ClinicalTrials.gov. A study evaluating the safety and efficacy of multiple treatment combinations in participants with multiple myeloma (PLYCOM). 2022. https://clinicaltrials.gov/ct2/show/NCT05583617.

[109] ClinicalTrials.gov. Study of ISB 1342, a CD38/CD3 bispecific antibody, in subjects with previously treated multiple myeloma. 2022. https://clinicaltrials.gov/ct2/show/NCT03309111.

[110] Madduri, D. REGN5458, a BCMA x CD3 bispecific monoclonal antibody, induces deep and durable responses in patients with relapsed/refractory multiple myeloma (MM). Blood. 2020;136:41–42.

[111] Zonder J. Early, deep, and durable responses, and low rates of cytokine release syndrome with REGN5458, a BCMA x CD3 bispecific monoclonal antibody, in a phase 1/2 first in human study in patients with relapsed/refractory multiple myeloma (RRMM). European Hematology Association (EHA) Oral Presentation S189 (2022).

[112] Cooper, D. Safety and preliminary clinical activity of REGN5458, an anti-BCMA x anti-CD38 bispecific antibody, in patients with relapsed/refractory multiple myeloma. Blood. 2019;136:3176.

[113] Zonder, J. Early, Deep, and Durable Responses, and Low Rates of Cytokine Release Syndrome withREGN5458, a BCMA x CD3 Bispecific Monoclonal Antibody, in a Phase 1/2 First in Human Study in Patientswith Relapsed/Refractory Multiple Myeloma (RRMM). Blood. 2021;138.

[114] Harrison, SJ. A phase 1 first in human study of AMG 701, an anti-B-cell maturation agent (BCMA) half-life extended (HLE) BiTE (bispecific T-cell engager) molecule, in relapsed/refractory (RR) multiple myeloma (MM). Blood. 2020;136;28–29.

[115] Krishnan A (2021). Updated phase I results from monumeTAL-1: first-in-human study of talquetamab, a g protein-coupled receptor family c group member 5 d x cd3 bispecific antibody, in patients with relapsed/refractory multiple myeloma. Blood.

